# The use of the World Health Organization Surgical Safety Checklist in operating theatres

**DOI:** 10.4102/hsag.v28i0.2246

**Published:** 2023-07-31

**Authors:** Mariet van Zyl, Neltjie C. van Wyk, Ronell Leech

**Affiliations:** 1 Department of Nursing Science, Faculty of Health Sciences, University of Pretoria, Pretoria, South Africa

**Keywords:** patient safety, World Health Organization (WHO), Surgical Safety Checklist (SSC), intraoperative teams, implementation science, qualitative research

## Abstract

**Background:**

There is a global concern over intraoperative patient safety, as adverse events are on the rise. When the World Health Organization Surgical Safety Checklist (WHO SSC) is used correctly, it has the potential to prevent such events. Unfortunately, the intraoperative team in the designated hospital lacked the cooperation to successfully use the checklist.

**Aim:**

This study, therefore, aimed to explore and describe the factors that affect the use of the checklist in the operating theatres in a designated hospital.

**Methods:**

A qualitative research approach together with an implementation science strategy structured according to the Consolidated Framework for Implementation Research was used. Individual interviews with nine surgeons and focus group interviews with six operating theatre professional nurses provided sufficient data for inductive and deductive analysis.

**Results:**

A deeper understanding of the contextual and interventional factors that affect the use of the WHO SSC is provided by the findings. A high demand for surgery, the hierarchy in the surgical team, their uncertainty about hospital policies and reluctance to adjust to change contributed to the poor use of the checklist.

**Conclusion:**

A sustainable implementation process is crucial and should be embraced and promoted by the intraoperative team.

**Contribution:**

The article contributes a description of the factors that address the use of a checklist for intraoperative patient safety. It recommends that the factors that hinder the use of the checklist be timeously addressed.

## Introduction

Surgical procedures are capable of producing adverse events, making operating theatres arguably one of the most challenging work environments in healthcare (Noaman, Soliman & Hasaneen 2020:116). The World Health Organization Surgical Safety Checklist (WHO SSC) was introduced in 2009, and intended to improve global intraoperative safety practices, as well as to reduce preventable adverse events (Hazelton et al. 2015:111). These safety practices may be beneficial to both patients and the intraoperative team (Erestam et al. 2016:2879). Despite the checklist being used on an international level, operating theatres still face challenges with sustainable implementation practices (Verwey & Gopalan 2018:341).

## Background

Globally patient safety is known to be one of the biggest challenges within the healthcare system. The World Health Assembly recognised these challenges and the need to improve healthcare quality (Woodman & Walker 2016:2). An international alliance was formed by 56 countries in 2004 to enable multi-disciplinary teams to develop guidelines for safe healthcare practices. The endeavour led to the WHO’s Patient Safety Campaign with the initiatives of ‘Cleaner Care is Safer Care’, and ‘Safe Surgery Saves Lives’ (WHO 2009:2–4).

Ten critical objectives for safe intraoperative care are outlined in the WHO Guidelines for Safe Surgery. The WHO SSC, which has been universally implemented since 2009, was founded on these objectives (Jain, Sharma & Reddy 2018:7). When the WHO developed the SSC, it had several goals in mind – including creating consistency in intraoperative care, establishing a safety culture, and encouraging adherence to an intraoperative safety culture (Georgiou et al. 2018:339). Ultimately, the aim of the WHO SSC is to ensure intraoperative safety for every patient undergoing surgery, by providing opportunities throughout the surgical procedure for the intraoperative team to collaborate and communicate effectively (Jain et al. 2018:7; Verwey & Gopalan 2018:336).

There are three components of the WHO SSC that require attention. All three components utilise different time intervals (Thomas et al. 2019:103) and address unique safety concerns intraoperatively. Firstly, ‘sign-in’ process component, where identification of patients and procedures is the foremost concern. During this time, any anaesthetic concerns may be addressed before the induction of anaesthesia starts. Secondly, the ‘time-out’ process component, which occurs after induction and immediately prior to the surgical incision. As a result of this process, the intraoperative team is able to re-evaluate and confirm the patient, the procedure details and associated risks, availability of needed equipment and results of diagnostic tests. This is the final safety check before the surgery commences and it is essential that the surgeon is present. Thirdly, ‘sign-out’ process component that occurs during or shortly after wound closure, but before the patient is removed from the operating theatre. The surgeon summarises the procedure that is just completed and the nurses verify that safe-counts were taken and that the results are accurate. The intraoperative team also reviews safety issues that need to be addressed for future surgeries in order to improve service delivery. To conclude the procedure, the postoperative care is discussed, and the patient is transferred to the recovery room (WHO 2009:99–105).

Although the WHO SSC implementation is proven to be simple, beneficial and cost-effective, sustainable implementation is often challenging (Delisle et al. 2020:151–152; Thomas et al. 2019:103). Controversy surrounds non-compliance with the use of the WHO SSC (Georgiou et al. 2018:340). Failure to implement in a successful way can compromise the safety of intraoperative procedures and the quality of patient care (Geerligs et al. 2018:1).

The first author (an intraoperative nurse for 20 years in the designated hospital) noticed that the intraoperative teams often did not use the checklist in the correct manner. The hospital management adjusted the guidelines and the checklist to fit the circumstances of its operating theatres and still, the team did not implement the WHO SSC as required.

According to implementation science, it is imperative to study the reasons why evidence-based guidelines such as the WHO SSC are not implemented (Nilsen 2015:1). The ‘science of how to most effectively promote and support the use of evidence in health and health care’ is referred to as knowledge translation, implementation science, quality improvement and dissemination (Colquhoun et al. 2014:1). In this study the authors refer to implementation science.

The aim of the study was to better comprehend the contextual and interventional facilitators and barriers in the use of the WHO SSC in the operating theatres of a designated hospital in South Africa.

## Methods

The study used a pragmatic paradigm, which puts aside philosophical disagreements in favour of what works in a certain situation or for a specific set of research questions while acknowledging diversity and complexity (Creamer 2018:45). A qualitative approach was adopted together with an implementation science strategy, which was structured according to the Consolidated Framework for Implementation Research (CFIR).

The implementation science methodology is described by Taylor, Bogdan and DeVault (2015:3–10) as a problem-solving approach. In implementation science, the problems that are encountered with the implementation of best practices are studied (Pinnock et al. 2017:1). Qualitative research methodologies can be used in implementation science to study the barriers and facilitators of the implementation of evidence-based practice (Palinkas et al. 2015:542). Tacit knowledge that people gain through experience and that enables them to know more than they can tell, is studied as it can be used to adapt best practices to enhance its relevance and applicability for the designated setting (Kothari et al. 2012:1). The guiding principles of qualitative research in implementation science can be leveraged by showing what questions are relevant, but they can also provide insights into problem-solving (Hamilton & Finley 2019:1).

Through qualitative research in implementation science, the intraoperative team got the chance to understand the complexity of the contextual and interventional facilitators and barriers that might have influenced the implementation of the WHO SSC.

### Sampling and setting

The study was conducted in a designated hospital in South Africa. The hospital provides access to the multidisciplinary intraoperative team, which formed the study population. The population consisted of 53 nurses and 46 medical practitioners (multidiscipline surgeons and anaesthetists). A criteria-i method of purposive sampling, which is well suited for qualitative research in implementation science, was performed. In qualitative research in implementation science, participants need to be selected ‘based on their level of participation in the implementation process’ (Colon-Emeric et al. 2016:3) that in this study refers to the use of the WHO SSC. The inclusion criteria referred to the use of the WHO SSC. The study sample included six operating theatre professional nurses and nine medical practitioners who were able to provide comprehensive descriptions of their perceptions regarding the factors that impact the use of the WHO SSC (refer to Table 1). Data saturation determined the number of participants.

**TABLE 1:** Democratic information of participants.

Type of interview	Participants	Designation	Function
Semi-structured focus group interview one	N1	Professional nurse	Scrub nurse
	N2	Professional nurse	Anaesthetic nurse
	N3	Professional nurse	Anaesthetic nurse
Semi-structured focus group interview two	N4	Professional nurse	Scrub nurse
	N5	Professional nurse	Anaesthetic nurse
	N6	Enrolled auxiliary nurse	Floor nurse
Semi-structured individual interviews	D1	Medical practitioner	Orthopaedic surgeon
	D2	Medical practitioner	Trauma surgeon
	D3	Medical practitioner	Urologist
	D4	Medical practitioner	Anaesthetist
	D5	Medical practitioner	Orthopaedic surgeon
	D6	Medical practitioner	Trauma surgeon
	D7	Medical practitioner	Urologist
	D8	Medical practitioner	Trauma surgeon
	D9	Medical practitioner	Trauma surgeon

### Data collection

An interview approach was deemed relevant for the data collection process. Semi-structured focus group interviews were conducted with the nurse participants and semi-structured individual interviews with the medical practitioner participants. One interview guide based on the domains of the CFIR was used in both types of interviews. The guide addressed the characteristics of the WHO SSC and the intraoperative team, the internal and external barriers and facilitators that could have influenced the use of the WHO SSC, and the process of using the WHO SSC. The interviews took place on dates and times prior arranged with the participants, in suitable venues. To prevent information to be lost during the interviews, participants allowed audial recordings of the interviews. Field notes were also recorded of data that were not included in the audio recordings. The interviews did not exceed 90 min.

### Ethical considerations

The University of Pretoria’s Faculty of Health Science Research Ethics Committee (Ethics reference no. 112/2020), gave their approval of the study to take place. The right to privacy of the participants and confidentiality of data shared with the researchers were upheld. Participation was voluntary and participants provided written informed consent prior to participation.

### Data analysis

Both inductive and deductive analysis methodologies were applied. The domains of the CFIR were used in the deductive analysis. In qualitative research in implementation science data are analysed according to frameworks (Breimaier et al. 2015:4). The data that does not fit the framework in implementation research should be thematically analysed in an inductive manner (Kothari et al. 2012:6). In the thematic analysis, a process was followed where the transcripts and field notes were read and re-read, and open coding was carried out to identify patterns in the data. The patterns were grouped to formulate common themes that were used to complement the findings derived from the deductive analysis (Colon-Emeric et al. 2016:3).

In the deductive analysis, the researchers applied the steps developed by Colon-Emeric and co-researchers (2016:3) who recommend that priori concepts be used to structure the analysis. In this study, the contextual and interventional factors as it is described in the CFIR were used. In implementation research, the priori concepts are mentioned in the research question, aim and objectives of the study (Colon-Emeric et al. 2016:3). In this study, it referred to the contextual and interventional factors that affect the use of the WHO SSC in the operating theatres in a designated hospital.

The deductive analysis was performed in five stages. In the first stage, the researcher familiarised herself with the data by reading and re-reading the transcripts and field notes. In the second stage, the CFIR domains and applicable constructs were used to code the data. The same document that had been used in the data collection was used as a framework in the data analysis to ensure that the priori concepts are included. In the third stage, the themes that had been formulated during the inductive thematic coding were incorporated into the codes that developed from the deductive coding. In the fourth stage, similar codes were grouped to form categories and sub-categories. Each sub-category was substantiated with excerpts from the transcripts. In the fifth stage, the categories and sub-categories were interpreted with references to the existing knowledge base. A comprehensive literature review was used to interpret the findings (Colon-Emeric et al. 2016:3).

The outcome of the inductive and deductive analyses was integrated in categories and sub-categories (refer to Table 2).

**TABLE 2:** Categories and sub-categories.

Categories	Sub-categories
**Domain: Outer setting**	
Category 1: Stakeholders’ influences on the WHO SSC implementation	Uncertainty about policies and legality of the WHO SSC Differences in expectations of professions
Category 2: Patients’ needs and expectations	High demand for surgery Utilisation of the WHO SSC addresses patients’ needs
**Domain: Inner setting**	
Teamwork is required for the WHO SSC implementation (Category 3)	Acknowledge and respect team members’ experience and responsibility Team characteristics that influence use of the WHO SSC Hierarchy and management are important elements that affect the WHO SSC implementation
Implementing the WHO SSC involves the use of communication infrastructure (Category 4)	Effective communication is needed Utilising existing structures to communicate
**Domain: Characteristics of individuals**	
Involvement and familiarity with the WHO SSC (Category 5)	Familiarity with the WHO SSC Individuals’ involvement in the current WHO SSC implementation
Confidence and capability of individuals (Category 6)	Confidence in participation Factors that affect confidence to implement the WHO SSC Perceptions of ability to effectively implement the WHO SSC Training to increase capability Acceptance of change
**Domain: Intervention characteristics**	
Developed by the WHO for patient safety (Category 7)	Aimed at prevention of harm to patients Does not prescribe responsible persons Associated with change
**Domain: Implementation process**	
Planning as a blueprint for implementation (Category 8)	Involvement in implementation planning process Effective discussions and training as part of the planning process
Importance of management involvement (Category 9)	Leadership and engagement methods
Effective execution of the checklist (Category 10)	Execution relies on adequate planning
Reflection in change management (Category 11)	Power of reflection in change management

*Source:* Van Zyl, M., 2022 ‘Contextual and interventional factors that affect the implementation of the World Health Organization Surgical Safety Checklist in operating theatres of a designated private hospital’, *Masters in Nursing Science dissertation*, University of Pretoria

### Trustworthiness

Methodological trustworthiness is crucial for building the body of knowledge in implementation science (Tracy 2010:840). The researchers consistently avoided biasness that could have affected the data collection and analysis. Triangulation was accomplished through interviews conducted with different members of the multidisciplinary intraoperative team. Focus group and individual interviews were performed. Both inductive and deductive data analyses methodologies were applied. The data’s transferability was achieved by a thorough description. The first author collected the data over a long period of time and thereby ensured prolonged engagement with the participants.

## Results

The results indicated that interventional and contextual facilitators and barriers influenced the implementation of the WHO SSC. The contextual factors are described under the following headings: Stakeholders’ influences on the WHO SSC use; Patients’ needs and expectations; Teamwork is required to use the WHO SSC; Involvement and familiarity with the WHO SSC; and Confidence and capability of individuals.

### Stakeholders’ influences on the World Health Organization Surgical Safety Checklist use

Different professional expectations and stakeholders’ uncertainties about policies and legality related to the WHO SSC are factors affecting the use of the checklist. According to the interview data, most participants were unaware of other policies, regulations or guidelines at local, state, and national levels, despite being aware of internal hospital policies and guidelines. The majority of participants believed that patient safety is a shared responsibility; however, a few participants believed that individual responsibility was necessary for the use of the WHO SSC:

‘No, I don’t know any local or national checklist or guidelines. There might be but it doesn’t come to my mind.’ (D1, Orthopaedic surgeon)

‘I feel um […], most of the times when the checklist is done, it is to protect the doctor and to protect us. But I feel the clients thinks it is only for us, it is our work that we want to force it on them. In fact, it is for both of us, the clients and the nursing staff.’ (N4, Scrub nurse)

### Patients’ needs and expectations

The use of the WHO SSC has the potential to enhance quality patient safety intraoperatively. There is, however, a risk of inadequate care and non-compliance to patient safety measures because of the high volume of surgical cases that need to be rushed through a long theatre list:

‘I think there are time constraints and the rush factor. We want to get patients in and out as quickly as possible. To limit theatre time, we tend to rush things […] and not do everything.’ (D4, Anaesthetist)

### Teamwork is required to use the World Health Organization Surgical Safety Checklist

Members of the intraoperative team work synchronously. The teamwork is often influenced by factors such as recognition of and respect for individual roles and experiences. The negative attitudes of authoritative team members may directly influence the use of the WHO SSC:

‘So, you have very senior surgeons, anesthetists, medical practitioners, who by force of habit do not practice the checklist […] and then we have the junior nurses who just don’t have the authority or the knowledge to enforce it.’ (D2, Trauma surgeon)

Various characteristics are needed to support effective and efficient teamwork in the use of the WHO SSC:

‘No. Some of them don’t want to do it. If you try and get involved, they don’t want you to speak on their behalf. They will stop you. And you get undermined quite often.’ (N2, Anaesthetic nurse)

The prevalence of hegemonic hierarchies often sacrifices the uses of the WHO SSC:

‘I would say that some staff fear the surgeon and is scared of the matron. And they tend not to question their decisions. If they say just put the patient on the table, they will not stop and say no we must do the checklist first. There is a hierarchy. And I will say there is definitely a fear factor. The staff are scared that the surgeon will scream at them or insult them or something.’ (D4, Anaesthetist)

### Implementing the World Health Organization’s Surgical Safety Checklist involves the use of communication infrastructures

According to the interview data, team effectiveness is dependent on communication. The effective use of communication and the utilisation of existing structures can play a substantial role in implementing the WHO SSC.

The implementation process of the WHO SSC is strained by a roller-coaster relationship between nurse’s negative attitudes and rudeness as well as the arrogance of doctors:

‘They give you a very negative attitude. Also, with some of the doctors, they are always in a rush, so they are rude and arrogant towards the nursing staff. Whereby they push the patient in quickly, and they are not interested in the nursing team doing the WHO SSC. It is a very […] uhm how can I put it […] a roller-coaster relationship.’ (N2, Anaesthetic nurse)

It was explained by participants that in the hospital, a variety of communication forums are available for discussing intraoperative process enhancements:

‘If the hospital wants to implement anything, they should present is at the doctors’ forum. The theatre forum with be based on the individual ones, because you can hardly get all the doctors there. Whereas if it starts at the doctor’s forum, it’s easier.’ (D8, Trauma surgeon)

### Involvement and familiarity with the World Health Organization Surgical Safety Checklist

The data showed that although the participants were aware of the importance of the WHO SSC, they were not very aware of the tool itself. Only few people knew that the WHO SSC is integrated in the hospital’s current perioperative documentation:

‘Currently I know that we should be using it, the WHO says we should. The organization has not communicated it specifically this WHO SSC very well. They did not say we going to use this, and this is what we want to use. They did not really communicate to me much, to the doctors.’ (D7, Urologist)

Familiarity with the WHO SSC facilitates understanding and increases willingness to participate in its use, according to the participants who answered a question on perceptions of participants’ participation in the process and their willingness and commitment to the process:

‘Yes, it will affect their willingness. If people are made to understand the importance of this, it will improve the efficiency and the safety of our patients.’ (D8, Trauma surgeon)

It still poses a challenge to successfully use the WHO SSC because of a lack of seniority among nurses, and the lack of interest among doctors who do not wish to participate:

‘Your problem is that the nursing sisters are not senior enough to ensure and insist that there is a force created. The surgeons and the anaesthetist do not take a particular interest, because they do not understand the requirement for it.’ (D2, Trauma surgeon)

### Confidence and capability of individuals

Confidence and capabilities of individuals are influenced by a variety of factors. One needs to consider: confidence in participation; factors that affect confidence to implement the WHO SSC; perceptions of ability to effectively implement the WHO SSC; training to increase capability; and acceptance of change. One participant said that knowing what needs to be done creates confidence and creates an awareness of safety processes:

‘The thing is, if you know something needs to be done, you have the confidence to do it. If you don’t think it is really necessary, then you just let it slide. And I think just being sure of what you are doing, will make you more aware of what needs to be done.’ (D4, Anaesthetist)

Guidance from more experienced team members can facilitate confidence in less confident members. This will assist in enhancing the use of the WHO SSC:

‘I think it becomes very individualised. I think that it is left to the people around to identify the new individuals and guide them through it. If you guide them through it, they will develop confidence and self-confidence in doing it.’ (D9,Trauma surgeon)

The participants believed that they should use the WHO SSC correctly; however, management’s support is needed for them to successfully and continuously use it:

‘Even if there is no interference from the doctors, I think it can be done effectively. And if the support is given from management, then it will actually be done continuously.’ (N5, Scrub nurse)

To facilitate team members’ understanding of why the WHO SSC is imperative, formal training is often necessary:

‘Because the people may not always know all the things that should be done. Insight will help people to understand it and implement it. It will also prevent people fighting it. A formal presentation will help, I think so.’ (D8, Trauma surgeon)

The participants felt that it is important to gain insight, be motivated, and be flexible in order for change to occur:

‘I think that insight, motivation, tolerance for change […] those are actually the most important attributes that in fact guide change, for someone to comply and be willing to change. Those are the pillars for change.’ (N5, Floor nurse)

The interventional factors are discussed under the following categories: developed by the WHO for patient safety; planning as a blueprint for implementation; importance of management involvement; effective execution of the use of the checklist and reflection in change management.

### Patient safety checklist developed by the World Health Organization

Patient safety is paramount in every aspect of healthcare, including the intraoperative environment. The WHO developed a SSC to improve intraoperative patient safety. It is aimed at prevention of harm to patients; it does not prescribe a responsible person and it is associated with change:

‘You are looking after the health of the patient and the treating staff of the hospital from the medical-legal point of view.’ (D5, Orthopaedic surgeon)

‘I think it is skimp over and done incorrectly. And it is done by the wrong people. I think that there should be a dedicated person allocated in theatre. Because, if everybody is responsible, then nobody is responsible.’ (D4, Anaesthetist)

‘And I feel that nurses are not doing surgical pause because of bad habits. Some came into the department saying others are not doing it, so why should I do it?’ (N4, Scrub nurse)

### Planning as a blueprint for implementation

Involvement in the implementation planning process and effective discussions and training as part of the planning process are factors that requires careful consideration to ensure that the intraoperative team uses the WHO SSC to improve patients’ safety:

‘It has to be teamwork. If you only leave it to one person the rest of the team would not know what to do, and if something happens the others won’t take responsibility for it. But if you, do it as a team, then everyone is responsible for it and they know what is going on and they can then account for it, the whole team.’ (N4, Scrub nurse)

‘Depends on the discussion. It depends on the style of the discussion. If you have an informal chat in the passage, it is practically useless. It needs to be formalised and supported by informal small group teaching. Large group VS small group teaching. In other words, you need both.’ (D2, Trauma surgeon)

### Importance of management involvement

Leadership and engagement factors play an important role in implementation processes. Constructive leadership and engagement of management are associated with positive results when initiatives such as the WHO SSC are implemented:

‘The managers, when you bring something new to the staff you need to do it by being hands-on. In that way you are leading and showing leadership. Implementation becomes easier.’ (N5, Anaesthetic nurse)

‘Management can’t be involved intra-operatively. So, the hospital management should say the theatre committees discuss this and implement this. No surgeon is going to refuse this. No anaesthetist is going to refuse this.’ (D6, Trauma surgeon)

### Effective execution of the use of the checklist

The factor coming to play here is the assumption that sufficient planning is needed to successfully execute the SSC. The results revealed that there was no formal approach or a designed plan to support and sustain the execution of the SSC:

‘But what was the plan? I don’t know.’ (N4, Scrub nurse)

‘There is no plan. There is no implementation.’ (D2, Trauma surgeon)

‘At this moment I think we should still wait and see. Currently I don’t think there is a plan.’ (D7, Urologist)

### Reflection in change management

Reflection is a powerful skill that is needed for development and enhancements of practices to enhance quality patient care. The results of the study indicated that reflection lays the foundation for improvements:

‘You got to look back on what you have done, to know how to go forward. If we don’t know how we are doing, we cannot devise a plan.’ (D3, Urologist)

## Discussion

In the context of high surgical demand, intraoperative teams are faced with a number of challenges. Best care practices and patient safety are directly affected by these challenges. As theatre lists fill up, and workloads increase, safety practices are neglected (Ayabe et al. 2019:24). Despite the hectic schedules of operating theatres, intraoperative team members often prioritise pushing procedures, sometimes at the expense of patient safety. Many theatre scheduling processes revolve around completing the sometimes overwhelming 12–15-h shifts without considering that each patient has unique needs and expectations. In addition to the fast pace, the team’s processes and relationships are stressed. Consequently, each of these factors impedes the use of the WHO SSC.

Intraoperative team collaboration becomes quintessential in a complex setting, where patient safety is dependent on multidisciplinary expertise (Etherington et al. 2021:2). Interdisciplinary teamwork requires mutual trust, respect and understanding of each other’s responsibilities to facilitate safety practices through the use of the WHO SSC.

Findings of this study revealed that the following characteristics are affecting teamwork as far as the use of the WHO SSC is concerned. Mutual respect and understanding in the team, interest in the use of the checklist, general support among team members as well as between the team and management, and effective communication can enhance the use of the WHO SSC. Members should understand each other’s roles and responsibilities to simplify communication between members and among the whole team. The member of the team is co-responsible for the use of the WHO SSC to enhance intraoperative patient safety.

Within the intraoperative environment, hierarchical power structures exist. Hegemonic hierarchies, poor cooperation of team members, overconfidence towards patient safety, together with indifference to the accountability and responsibility of teams, are detrimental to intraoperative teams’ use of the WHO SSC to secure patient safety. When hierarchy attracts more focus than safety practices, intraoperative safety suffers (Weinger 2021:2).

Effective communication depends on correctly framed sentences and appropriate intonation. Besides ensuring that the message is understood correctly, the interpretation process also stimulates team interaction (Choi, Park & Kim 2019:1–2). In the context of theatre forums and physician advisory boards, effective communication can facilitate familiarising key stakeholders with the WHO SSC and its implementation processes. As leaders in their fields, they can gradually influence cooperation with other stakeholders through their support and buy-in. Communication quality is important, not quantity (Marlow et al. 2018:2), in a truly diverse and dynamic team. An intraoperative team’s collaborative effort is what ensures the safety of patients. Communications structures exist, but are not effectively utilised. This results in inefficient stakeholder engagement. Inefficiency in management teams can lead to a delay or total rejection of proposed interventions. Medical and nursing teams can achieve greater cooperation and trust if these platforms are used properly, improving the rate of acceptance of interventions (Lombardi et al. 2018:8).

Intraoperative patient safety is concomitant with physical, intellectual, and professional challenges (Pavlová, Holá & Škaroupková 2019:1125). Intraoperative teams are aware of the risks and benefits associated with the use of the WHO SSC for patient safety. The WHO SSC cannot be understood by merely knowing about it. Even if team members are aware of the WHO SSC and its benefits, if they do not know the actual tool and cannot apply it, the effort will be futile. Increased intraoperative safety risks and reduced operational efficiency are caused by ignorance of the benefits of the use of the WHO SSC (Greig, Maloney & Higham 2020:304). Safety processes in the intraoperative context are not isolated actions. Omissions to comply by one person impacts the implementation process and effective team collaboration. Factors such as fear, anxiety and poor support systems, contribute to irregularities and non-compliance.

When experienced operating theatre professional nurses start to initiate each section of the WHO SSC, intraoperative teams will become more accustomed to using it. Experienced operating theatre professional nurses understand the importance of collaboration within the entire surgical team (Gong et al. 2021:5). They should engage and familiarise all team members with the use of the WHO SSC.

The participants reflected on different factors, which impact confidence within the intraoperative context. Instilling confidence in one’s abilities is a personal journey that is influenced by outside factors. Developing a sense of confidence, facilitates understanding the methods and processes for using the WHO SSC. Confident individuals can cooperate with other team members to improve the efficiency of the team. Positive correlations exist between work confidence and willingness to work. While it is imperative to maintain confidence and efficacy, not everyone is confident of their ability to initiate and sustain the use of the WHO SSC. The participants were convinced that being part of a functional team, effective communication between team members, a culture of teaching and learning, and a relaxed working environment increased their confidence. A lack of confidence and efficacy hinders the use of the WHO SSC, which divert attention from patient safety to creating opportunities for errors to occur. The maintenance of a patient safety culture requires change management and the involvement of all people involved. Individuals should be prepared to accept the change and be willing to adjust to it (De Jager, Gunnarsson & Ho 2019:120). The successful implementation of change is time-consuming and requires that all team members should embrace it (Nordström & Wihlborg 2019:224). The intraoperative team members need to first accept that the use of the WHO SSC may bring about change in their routine practices. Whether they view the use of the WHO’s SSC as a means to enhance patient safety depends on individual perspectives (Delisle et al. 2020:157). It is crucial to have adequate training to accept and implement change. The participants believed that negative perceptions about the contribution that the use of the WHO SSC can make towards patient safety arise from a lack of training.

In the absence of acknowledgement of these factors, it may act as barriers to the use of the WHO SSC. It is possible to reduce risks to patient safety intraoperative through the use of the WHO SSC. The use of the checklist can be initiated by a member of the team even though the WHO did not suggest a specific responsible team member such as the surgeon or operating theatre professional nurse (WHO 2009:7). The maintenance of the use of the WHO SSC is the responsibility of the team (Greig et al. 2020:304). It is therefore important that the whole team should take responsibility for the use of the WHO SSC (Willassen, Jacobsen & Tveiten 2018:8). Integrating the WHO SSC into the daily intraoperative routine promotes team collaboration and the creation of a safety culture (Weiser & Haynes 2018:297).

Formal implementation planning provides team members with standards, processes and training to adjust to the prescribed change, facilitate team collaboration to accept the change, and allowing opportunities to the team to take ownership of the change. During the planning phase, the focus should be both practical and theoretical to enhance buy-in by the team members. By involving all people involved in the implementation of the change the development of hegemonic hierarchies is prevented, cultural diversity gets managed and open, honest and respectful discussions are supported (Botchwey & Umemoto 2020:333–335). Buy-in and understanding from team members are crucial during the planning process to reduce barriers to the implementation. Team members share their perspectives during the planning phase, and as a result, foster mutual learning (Kaasinen et al. 2019:4). A lack of competence and insufficient training can lead to ineffective leadership to drive the use of the WHO SSC. It becomes a barrier to the use of the WHO SSC. Through extensive consultation and collaborative planning and implementation beneficial outcomes become possible (George, Walker & Monster 2019:812).

Complexed intraoperative environments necessitate the need for constructive and supportive multidisciplinary leadership. Knowledgeable managers do not only facilitate team collaboration but also encourage commitment to the execution of the initiatives such as the use of the WHO SSC (Hellar et al. 2020:694). Professional commitment forms the cornerstone of professional competence (Jafaraghaee et al. 2017:1).

When a plan is absent, or the planning is ineffective, it has a negative impact on the process of implementation (Zytoon 2021:77290), which creates resistance to change, especially among the more senior professionals (Tostes & Galvão 2019:9). In this study, the participants conveyed their concerns that the senior members of the intraoperative team were reluctant to use the WHO SSC to enhance patient safety.

Ongoing reflection regarding the facilitators and barriers that influence the use of the WHO SSC can provide valuable insight that can be beneficial for future endeavours to enhance patient safety (Sibbald et al. 2021:2). Through timeous reflection the quality of intraoperative care can be sustained (Murphy, Franz & Schlaerth 2018:1).

### Study’s limitations

The use of criterion-i purposive sampling for this study, involving just one hospital, may have resulted in a sample that seems not to be representative of intraoperative teams in general.

## Conclusion

A designated hospital in South Africa’s Gauteng Province is facing challenges in implementing the WHO SSC in operating theatres. The intraoperative team appeared to be uncertain about the hospital policy regarding the implementation of the WHO SSC and the high demand for surgery also contributed to the poor implementation of the checklist. The team did not agree on who should be the responsible person for the use of the checklist and the professions did not agree on how the checklist should have been used. Power struggle in the team aggravated the confusion about the use of the checklist. The team requires training in adjusting to change.

### Implications and recommendations for practice

The maintenance of intraoperative patient safety necessitates the integration of the use of the WHO SSC into daily practices. To ensure patient safety, intraoperative teams must become familiar with both external and internal policies that may influence the use of the WHO SSC. Communicating with all team members as well as the hospital management can prove beneficial not only in introducing them to the WHO SSC but also in gaining insights from them. Training sessions, both formal and informal are evidently necessary. The WHO SSC must be applied correctly in order to enhance patient safety.
